# Constructing three-dimensional (3D) nanocrystalline models of Li_4_SiO_4_ for numerical modeling and simulation

**DOI:** 10.1038/srep10698

**Published:** 2015-06-02

**Authors:** Yanhong Shen, Tao Gao, Xiaofeng Tian, Xiaojun Chen, ChengJian Xiao, Tiecheng Lu

**Affiliations:** 1 Institute of Atomic and Molecular Physics, Sichuan University, 610065, Chengdu, China; 2 Key Laboratory of High Energy Density Physics and Technology of Ministry of Education, Sichuan University, Chengdu, 610064, China; 3 Institute of Nuclear Physics and Chemistry, China Academy of Engineering Physics, Mianyang 621900, China

## Abstract

The three-dimensional (3D) nanocrystalline models of lithium silicates with the log-normal grain size distribution are constructed by constrained Voronoi tessellation. During evolution process, the algorithm is improved. We proposed a new algorithm idea by combining Genetic Algorithm (GA) with Least Square (LS) method to make up for the disadvantages of traditional genetic algorithm which may be easily trapped in local optimal solution. In the process of modeling, it is the first time, to the best of our knowledge, that we keep the whole sample showing the charge neutrality by deleting the excess atoms on the polyhedron boundary during the modeling. By using the molecular-dynamics method, the relaxation procedure of nanostructured Li_4_SiO_4_ is carried out. The results show that the average mass density of the sample is slightly lower than the experimental data of the perfect crystal after relaxation process. In addition, boundary component proportion (BCP) and density reduction proportion (DRP) of the sample is obtained, respectively. The present results display a significantly reduced BCP but an increased DRP when increasing the mean grain size of the sample.

With the decrease of the fossil energy, the improvement of mankind energy demand year by year. International Thermonuclear Experimental Reactor (ITER plan) project, which is also referred to as man-made sun, is an important plan to solve the energy problem. Recently, the use of solid breeding material is a promising option to breed tritium safely and economically, and ternary lithium- containing oxide Li_4_SiO_4_ with high tritium generation and fast tritium release has been considered as the first candidate for tritium breeding materials in ITER test blanket module (TBM)^1^,^2^,^3^. In fact, for a better of understanding tritium release, Carrera *et al.* investigated tritium recovery from nanostructured LiAlO_2_ which was also a tritium breeding material by X-ray diffraction and electron microscopy and indicated superlattice nanostructure may modify tritium release^4^. Therefore, it will be interesting to investigate the nanostructural features of the tritium breeding materials.

Even though there exists a number of experimentally and theoretically for the intrinsic structure, effective thermal conductivity, lattice dynamical, optical and thermodynamic properties of Li_4_SiO_4_ using different methods^5^,^6^,^7^,^8^,^9^, its many characteristics have still not been researched sufficiently, such as, diffusion constants of tritium, the mechanism of tritium release, the generation condition of the defects, and so on. Indeed, as the experimental capabilities for investigating the characteristics under dynamic conditions are extremely limited, it is an urgent need for modeling techniques of nanocrystals which can permit a wider and deeper understanding of some of the effects have been observed and may help to explain the mechanism of the phenomena.

Several methods have been proposed to generate digital nanostructured materials for use in the prediction of properties from microstructure. Such as, Monodispersive grain size (MGS) model^10^, Poisson–Voronoi tessellation (PVT) model^11^, Laguerre–Voronoi tessellation (LVT)^12^, Johnson–Mehl(JM) model^13^, and so on. According to the experimental grain size, shape and orientation statistics in two orthogonal sections through an aluminum polycrystal, Saylor *et al.* used a voxel-based tessellation technique to create a statistically representative 3D model microstructure for input into computer simulations^14^. Gross *et al.* used the inverse Monte Carlo method to build the microstructures of poly and nanocrystalline materials^15^. By utilizing the genetic algorithm, Tomoaki *et al.* developed a method to numerically construct a polycrystalline structure with a specified grain size distribution^16^. L. St-Pierre *et al.* applied Voronoi tessellation method to construct the 3D simulations of microstructure, using TiAl and grade 702 zirconium as the sample materials^17^. Although their models, to some extent, reflected some characteristics of nanocrystalline materials, they had not presented a good solution method for the complex structural nanocrystals. To data, it is generally accepted that many single-phase fully dense nanocrystals are described by a log-normal grain size distribution^14^,^15^,^16^,^17^. Therefore, by using the improved algorithm based on the genetic algorithm combined with least square method, the 3D models of nanostructured Li_4_SiO_4_ with the log-normal grain size distribution have been built successfully in this work.

## Results and Discussion

### Construction of an initial nanocrystal

**Figure 1:**
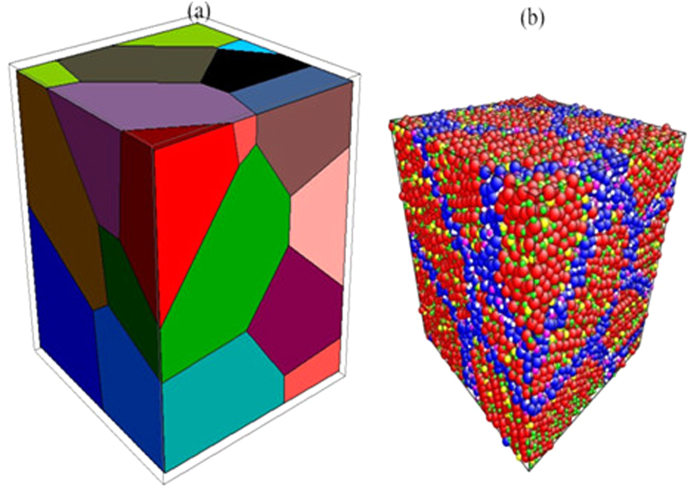
(**a**) The constructed box. (**b**) The unrelaxed atomic structure of a Li_4_SiO_4_ nanocrystal. (The color of Li, Si and O in the crystal atoms is red, yellow, and green, respectively. The color of Li, Si and O at the boundary atoms is blue, pink, and white, respectively; r_Li_ > r_Si_ > r_O_).

As we know, the microstructure plays an important role in determining various properties of nanocrystals. This work uses Li_4_SiO_4_ with monoclinic structure as a model material and provides a new method to simulate the atomic structural properties of the sample. The algorithm we developed is described as follows:

(1) Construct an orthogonal box with volume V_1_, and randomly generate N points or grains within the box;

(2) The Voronoi tessellation using these center points is built and the size-distribution 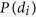 and the penalty function W^2^ (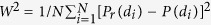) are computed. For improving the quality of solutions and reducing execution time, we introduce the genetic algorithm method combined with the least square method instead of the inverse Monte Carlo method of Gross *et al.*^15^. and the Genetic Algorithm of Tomoaki *et al.*^16^. The entire process repeats many times until W^2^ approaches the optimization criterion at W^2^ <10^-6^. Finally, we need retain these ultimate points which meet the requirements of W^2^ <10^-6^, and rebuild Voronoi polyhedron cells around each of these ultimate points.

(3) When using the atoms to fill the orthogonal box, we select the volume V_2_, according to a certain proportion, from the entire box (V_1_) to fill atoms in order to observe the structure of nanomaterial more clearly (see Fig. 1a). Generate a big enough supercell of Li_4_SiO_4_ with the volume V_3_ which should contain the circumscribed sphere of the volume V_2_ (V_3_ > V_2_); Move the supercell and make the center of the supercell and the center of the new box with the volume V_2_ overlap;

(4) Select the supercell’s rotation axis randomly and rotate the supercell according to arbitrary rotation angle. Fill one of Voronoi polyhedron cells in the new box with qualified atoms in the supercell. The qualified atoms need meet the following conditions^18^: The distance between the atom and the edge of a polyhedron is smaller than BDV (Boundary Displacement Variable) = βr_0_, (β ≥ 0, r_0_ is the atomic radius), while the nearest distance between two atoms on the two adjacent polyhedra is set no less than the diameter of the atom according to the idea of the hard-sphere model.

(5) The fourth step is repeated until all polyhedra in the new box are filled.

(6) Delete the excess atoms on the polyhedron boundary in order to keep the whole nanocrystalline material showing the charge neutrality (see Fig. 1b). At the same time, three-dimensional periodic boundary condition for the large-block is adopted, which will make the block effectively infinite and eliminate the influence of the boundary on the simulation results.

### Mean Grain Size Distribution

**Figure 2:**
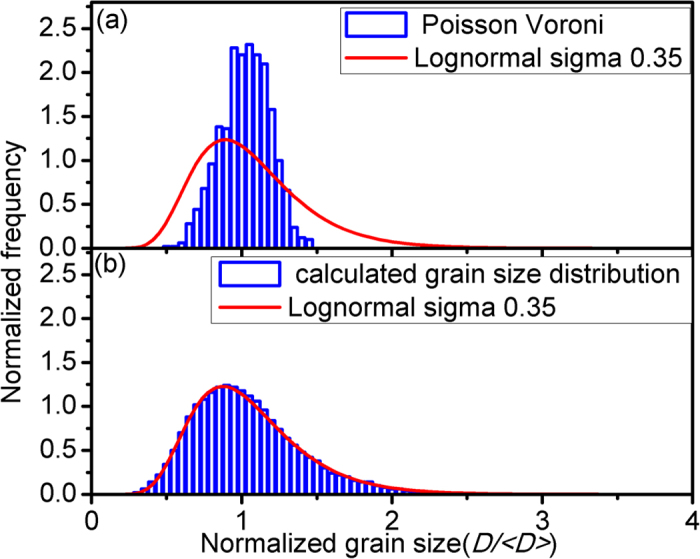
(**a**) The mean grain size distribution of the 3D model of nanomaterial with 1000 grains at the beginning of the algorithm is represented by the histogram. (b) The calculated mean grain size distribution of the 3D model of nanomaterial with 1000 grains after 10000 steps of evolution is represented by the histogram. The red line indicates the desired log-normal size distribution with σ = 0.35 and the fitted size-distribution of the histogram has σ = 0.34.

Among the simulation variables, the mean grain size (D) is the most important one because it direct controls the volume fraction of the boundary atoms. By using the new stochastic search and geometric computation, the mean grain size distribution of the 3D nanocrystalline model of Li_4_SiO_4_ with 1000 grains is obtained (see Fig. 2). The targeted grain size distribution is a log-normal grain size distribution with a variance of (0.35)^2^, the same as reported in Refs^15^,^16^.

During evolution process, we improve the algorithm by combining Genetic Algorithm (GA) and Least Square (LS) method to produce better quality results in smallest amount of time. The GA is a kind of global optimal searching algorithm based on Darwin’s nature evolution theory and Mendel’s genetics and mutation theory. The conceptualisation of GA in current form is generally attributed to Holland^19^. Compared with any other optimization algorithms, the outstanding excellences of GA are the capability of global optimization, strong robustness and inherent parallelism. However, GA has the shortcoming that it is easy to trap at local minima and time-consuming to find an optimal solution^20^,^21^. Therefore, we introduce the Least Squares (LS) method in this work. It is known to all that LS is a very good mathematical optimization technique which is usually used to find the best match solution by minimizing the error value between actual data and calculated data. Moreover, the volume of each Voronoi Cell is calculated by Voro++which is a software library for carrying out three-dimensional computations of the Voronoi tessellation^22^.

**Figure 3:**
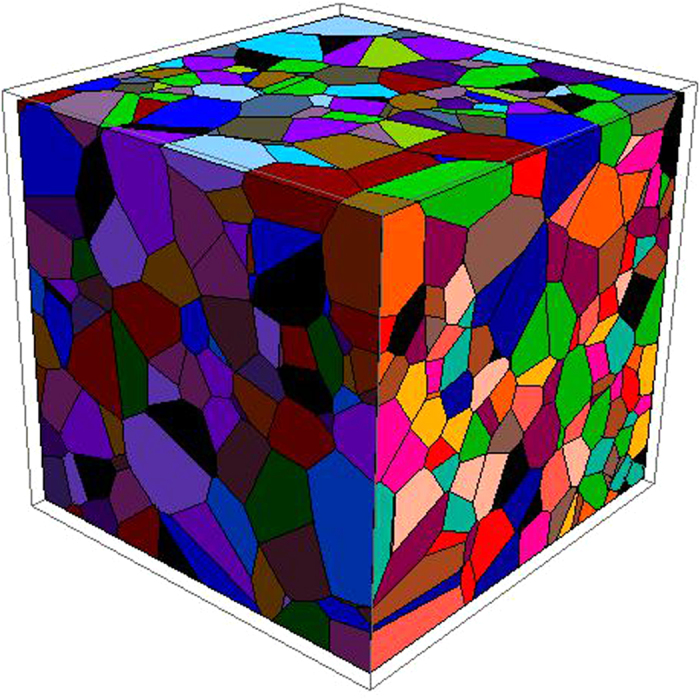
A 3D view of a nanocrystalline structure with 1000 grains created by the improved algorithm.

In Fig. 2, it can be easily seen that the mean grain size distribution of the sample obey the log-normal grain size distribution almost perfectly after 10000 steps of evolution, which is better than the results in Refs^15^,^16^. In this study, we adopted a population size of 32, the same as the number of population in Ref^16^. The computation time per step was 3.7 s with 6 processors, which has a higher efficiency than that of 3.9 s with 32 processors^16^. The mutation rate per center point is set at 0.1 for the first 500 generations, 0.01 for 500 ~ 1000 generations, and 0.001 for the rest. By applying the new method, we can give priority to a certain part of the mean grain size distribution function which matches worst with the standard log-normal distribution. During the study, we use a new fitness function F instead of the original fitness function W^2^ (or 1/W^2^) to help to escape local optimal solution efficiently. F is defined as: 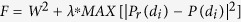;(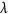 is an arbitrary constant and 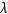 > 0). Thus, when the value of W^2^ has the same value, the result will give priority to the influence of the value of 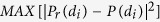. The smaller the value of 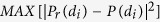 is, the better the value of F is. By utilizing the new fitness function F, we improve the quality of solutions and reduce execution time. Fig. 3 presents a 3D view of a polycrystalline structure with 1000 grains created by the new method.

### Relaxed Atomic Structure

Computer simulation is a powerful method to obtain the numerical approximation of microstructural changes taking place in nuclear materials. In fact, in the process of deleting the excess atoms on the polyhedron boundary, some artificial defects and torsions at the grain boundary is inevitably introduced, such as, the existence of the vacancy, the mismatch of ionic bonds, tilt boundary, and so on. For investigating the atomic position change and eliminate the influence of the artificial defects and torsions at the grain boundary of nanostructured Li_4_SiO_4_, the relaxation procedure was performed with the LAMMPS code^23^, based on the molecular-dynamics (MD) theory.

In this work, the primitive cell of Li_4_SiO_4_ has a monoclinic structure with space group of P2_1_/m (No.11)^24^. The atomic potential of Li_4_SiO_4_ used in this work, which is the pair potential function, is from the studies of Takahashi *et al.*^25^. In physics, a pair potential is a function that describes the potential energy of two interacting objects. Hence, we have adopted the same pair potential within crystal and among crystals. The pair potential functions are assumed to consist of simplified coulombic and repulsive terms: 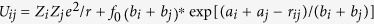, where 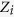 and 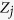 represent ionic charges, respectively, 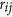
[Å] is the distance between the ions i and j, a and b are the parameters related to the radius and the compressibility of each ion, respectively, 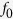 is an arbitrary constant (taken to be 6.7472*10^−11^N). The parameters used in the calculation are given in Table 1.

To perform the simulations, the cutoff distance for the Takahashi’s potential r_c_ was set to 10 Å. The coulombic term was evaluated using the Particle–Particle Particle-Mesh (PPPM) method^27^, while the repulsive term was described by a Buckingham potential. The Periodic Boundary Conditions (PBC) was employed. As the starting configuration, the simulation was run for 30,000 time steps (30 ps) in an NPT ensemble at zero external pressure to ensure that the system have adequate time to obtain a suitable equilibrium structures after relaxed process. In the process of MD run, the orthogonal box was flexible. The system was thermalized at 300 K. A time step of 0.001 ps was used in the MD runs. Finally, the system reached the equilibrium state because the fluctuation range of its temperature or energy was not very intense. Although this work has focused on Li_4_SiO_4_, the technique described here is general and it can be applied equally to any nanomaterial for which its atomic potential is available.

**Figure 4:**
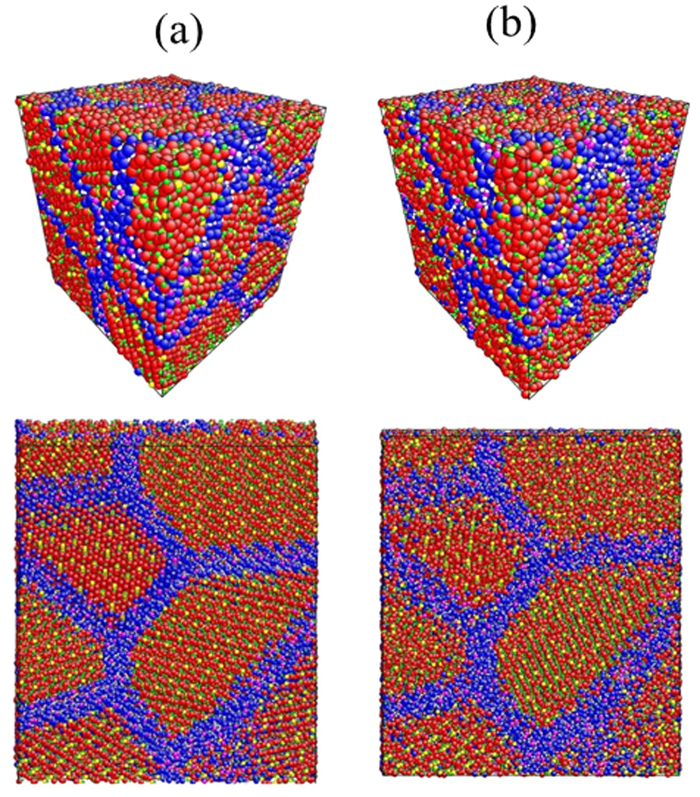
(**a**) The unrelaxed initial structure of a Li_4_SiO_4_ nanocrystal. (**b**) The relaxed atomic structure of a Li_4_SiO_4_ nanocrystal. (Mean grain size D=6.61 nm, BDV=0.5r_0_, where r_0_ is the radius of the Li atom).

The unrelaxed and relaxed atomic structures of nanostructured Li_4_SiO_4_ are illustrated in Fig. 4. As we can see, in Fig. 4(a), the atoms are arranged in an orderly state, while the arrangement of atoms is in the messy state in Fig. 4(b). Firstly, the phenomena may be caused by the change of the positions of the atoms which will move to the positions with minimum energy in order to achieve the steady state after the relaxation process. Secondly, the shape of the nanocrystal may also affect the simulation results as mentioned in Barnard *et al.*‘s work^28^,^29^.

In Fig. 4(a), the whole system is in a metastable state. With the development of the relax process, it will experience the transition from metastable state to steady-state. Finally, the whole system will reach the equilibrium state (see Fig. 4b). After relaxation process, the calculated bond distances of Li-Li, Li-O and Si-O of Li_4_SiO_4_ within the crystal are about 2.373–2.619 Å, 1.865–2.478 Å and1.589–1.712 Å, which are very close to 2.385–2.595 Å for Li-Li, 1.863–2.457 Å for Li-O and 1.597–1.696 Å for Si-O in the perfect crystal of Li_4_SiO_4_^30^, respectively. However, the bond distances between atoms at the grain boundary are not with certain regularity properties. By and large, although the atomic positions of nanostructured Li_4_SiO_4_ change after relaxation process, the bond distances between atoms within the crystal have good consistency with the uniform distributed crystal system.

In addition, the sample reveals a reduction in the atomic densities after relaxation process. In Table 2, the structural information of 3D model of the sample after relaxation process is given by the AtomEye^31^. It’s easy to observe that the stoichiometry is conserved and the ratio of Li: Si: O is still 4:1:4 in the final nanocrystal (see Table 2). The results also show that the average mass density of the sample is slightly lower than the experimental data of the perfect crystal^32^, which is a normal phenomenon because of the presence of the grain boundary atoms. Similarly, the phenomenon was discovered by Herr *et al.*^33^. using nanocrystalline Fe as the sample material.

### Boundary Component Proportion (BCP)

In order to illustrate the validity of the numerical simulation and modeling, we calculate the boundary component proportion (BCP) of nanostructured Li_4_SiO_4_ after relaxation process. (Where BCP=N_b_/N_t_, N_b_ is the number of boundary atoms, N_t_ is the total number of atoms in the nanostructured Li_4_SiO_4_.)

**Figure 5:**
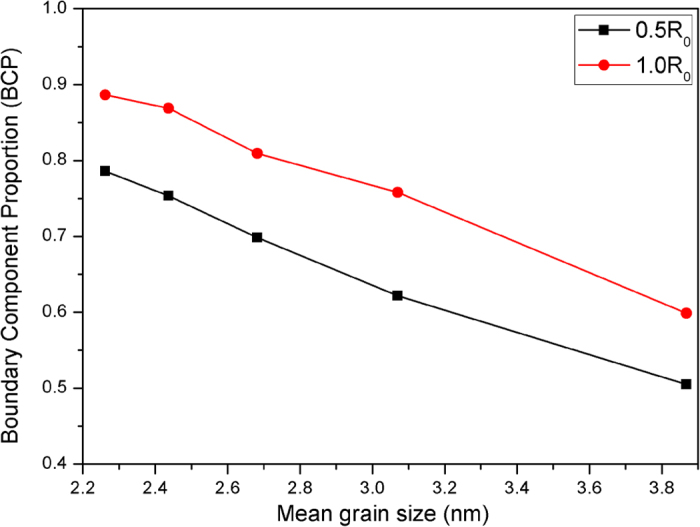
Boundary component proportion (BCP) of nanostructured Li_4_SiO_4_. The black and red line corresponds to the variable of BDV=0.5r_0_ and BDV=1.0r_0_, respectively.

In Fig. 5, we observe that the values of the boundary component proportion are very high (0.51–0.78). All the BCP’s are higher than 0.5, which means more than 50% of the atoms are situated in the boundary region. Compared the results of our calculations with the theoretical calculation of Chen^18^, it is of interest to note that our results display very good agreement with the values of Chen who mainly focused on the model material for the Fe nanocrystal. On the experimental side, Wallner *et al.*^34^. also observed that the mean atomic density in the interfaces was about 0.52 of the lattice density by utilizing the small angle scattering of neutrons and X-rays.

Furthermore, we observe that the values of the BCP present a declining trend as the mean grain size is raised (see Fig. 5). The change of the BCP in our calculations for the sample may be induced by the low boundary density and the enlarged interatomic spacing at the boundary region as the mean grain size of the sample is increased. The atomic structure of an interface depends on the orientation relationship between adjacent crystals and the boundary inclination^18^. With the elevation of the mean grain size of the sample, the interatomic spacing at the boundary region will enlarge due to the considerable misfit between adjacent crystals. A low boundary density and a high interatomic spacing will naturally lead to the decrease of the BCP in the nanostructured Li_4_SiO_4_.

### Density Reduction Proportion (DRP)

For the purpose of studying the changes of the Li_4_SiO_4_ nanocrystal which is induced by a low boundary density, the reduced density proportion (RDP) of the Li_4_SiO_4_ nanocrystal are computed (Where RDP=N_t_ / N_b_, N_b_ is the number of atoms in a perfect crystal with the same size as the nanostructured Li_4_SiO_4_).

**Figure 6:**
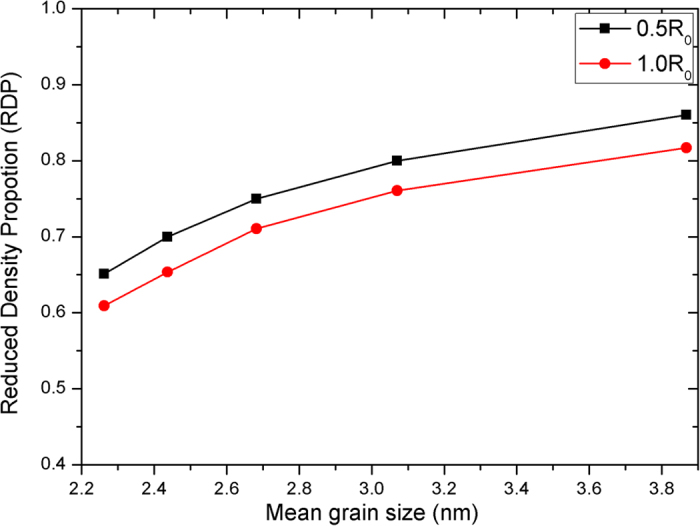
Reduced density proportion (RDP) of nanostructured Li_4_SiO_4_. The black and red line corresponds to the variable of BDV=0.5r_0_ and BDV=1.0r_0_, respectively.

As can be seen from the formula, the larger the value of DRP is, the higher the actual density of the sample is. Fig. 6 displays that the values of RDP vary between 0.65 and 0.87. We compare our calculated results with the recent theoretical and experimental data available, we find that our results is consistent with the calculated values by Chen (0.61–0.81)^18^ and the experimental results (0.6–0.9) under the different materials and experimental conditions^35^. That is to say, our method in this work is reliable and reasonable.

The above results may be attributed to the following reasons: On one hand, with the mean grain size increasing, the number of grains (N) in the same volume will decline. So the degree of misfit between the different grains will also decrease. On the other hand, an important effect is also worthy of our consideration that the results may be affected by the number of random displacement atoms at the grain boundary in the simulation. With increasing of the mean grain size, the number of the allowed random displacement atoms at the grain boundary will become small. Finally, when increasing the mean grain size of the sample, these factors will result in a lower value of BCP but a higher value of DRP as shown in Fig. 5 and Fig. 6, respectively.

By comparing our nanostructured 3D models as well as the nanocrystalline structures directly from experimental observations, such as electron backscatter diffraction (EBSD)^17^ or synchrotron radiation tomography^36^, our method is much more cost-effective and takes less time, which also proves its great potential value in applications for the development of nanocrystalline materials. The simulation demonstrates that the initial microstructure is very important because it depends sensitively on the methods used. Moreover, one innovative feature of the program developed is the possibility to generate 3D microstructures for the large-scale atomistic simulations and complex nanostructured modeling. Currently, the numerical modeling constructed using the new method is under way for heat transmission, mechanical properties, defect researches, surface microstructures, and transport properties of nanostructured Li_4_SiO_4_. The extension of the evolutional approach is capable of improving the efficiency of numerical computations largely. Hence, we hope that the method we developed here may potentially open a new path way for guiding the further theoretical researches and experimental explorations of nanocrystalline materials.

## Methods

A simplified overview of the actual process is shown in the diagram below. BEGIN (GA and LS) Create initial population (p1) randomly Calculate the value of W^2^ of each individual in p1 While Min(W^2^) > 10^−6^, DO BEGIN Produce new population (p2) from initial population (p1) (The two types of   mutations are considered: a new center point is selected completely   randomly; and it randomly shifts a short distance) Determine dynamically the fitness function F by individuals in p1 and p2 Select survival individuals from p1 and p2 by the new fitness function F Merge survival individuals in p2 and p1 as p1 Calculate the value of W^2^ of each individual in p1 END Output the individual END

## Additional Information

**How to cite this article**: Shen, Y. *et al.* Constructing three-dimensional (3D) nanocrystalline models of Li_4_SiO_4_ for numerical modeling and simulation. *Sci. Rep.* **5**, 10698; doi: 10.1038/srep10698 (2015).

## Figures and Tables

**Table 1:** Potential parameters used in the MD calculations.

**Parameters**	**Atoms**		
	**Li**	**Si**	**O**
a^a^	0.930	1.012	1.629
b^a^	0.080	0.080	0.085
Z^b^	+1	+4	-2

^a^Ref 25

^b^Ref 26

**Table 2:** The AtomEye shows the structural information of 3D model of a Li_4_SiO_4_ nanocrystal after relaxation process.

**Type**	**Mass(aum)**	**Count**	**Abundance**	**Wt.pct.**	**Avg. mass density(g/cm**^**3**^)	**Exp. mass density(g/cm**^**3**^)^a^
Li	6.941	29408	44.44%	23.17%	2.29	2.42
O	16.000	29408	44.44%	53.40%		
Si	28.088	7352	11.11%	23.44%		

^a^Ref 31
